# Differential virus host-ranges of the *Fuselloviridae* of hyperthermophilic Archaea: implications for evolution in extreme environments

**DOI:** 10.3389/fmicb.2012.00295

**Published:** 2012-08-24

**Authors:** Ruben M. Ceballos, Caleb D. Marceau, Joshua O. Marceau, Steven Morris, Adam J. Clore, Kenneth M. Stedman

**Affiliations:** ^1^Native American Research Laboratory, Division of Science and Mathematics, The University of MinnesotaMorris, MN, USA; ^2^Department of Biology, Center for Life in Extreme Environments, Portland State UniversityPortland, OR, USA; ^3^Department of Microbiology and Immunology, School of Medicine, Stanford UniversityStanford, CA, USA; ^4^Department of Biomedical and Pharmaceutical Sciences, The University of MontanaMissoula, MT, USA

**Keywords:** Archaea, Crenarchaea, *Fusellovirus*, halo assay, host-range, hyperthermophilic, *Sulfolobus*, *Sulfolobus* spindle-shaped virus

## Abstract

An emerging model for investigating virus-host interactions in hyperthermophilic Archaea is the *Fusellovirus-Sulfolobus* system. The host, *Sulfolobus*, is a hyperthermophilic acidophile endemic to sulfuric hot springs worldwide. The Fuselloviruses, also known as *Sulfolobus* Spindle-shaped Viruses (SSVs), are “lemon” or “spindle”-shaped double-stranded DNA viruses, which are also found worldwide. Although a few studies have addressed the host-range for the type virus, *Sulfolobus* Spindle-shaped Virus 1 (SSV1), using common *Sulfolobus* strains, a comprehensive host-range study for SSV-*Sulfolobus* systems has not been performed. Herein, we examine six *bona fide* SSV strains (SSV1, SSV2, SSV3, SSVL1, SSVK1, SSVRH) and their respective infection characteristics on multiple hosts from the family *Sulfolobaceae*. A spot-on-lawn or “halo” assay was employed to determine SSV infectivity (and host susceptibility) in parallel challenges of multiple SSVs on a lawn of a single *Sulfolobus* strain. Different SSVs have different host-ranges with SSV1 exhibiting the narrowest host-range and SSVRH exhibiting the broadest host range. In contrast to previous reports, SSVs can infect hosts beyond the genus *Sulfolobus*. Furthermore, geography does not appear to be a reliable predictor of *Sulfolobus* susceptibility to infection by any given SSV. The ability for SSVs to infect susceptible *Sulfolobus* host does not appear to change between 65°C and 88°C (physiological range); however, very low pH appears to influence infection. Lastly, for the virus-host pairs tested the *Fusellovirus-Sulfolobus* system appears to exhibit host-advantage. This work provides a foundation for understanding Fusellovirus biology and virus-host coevolution in extreme ecosystems.

## Introduction

**Figure 1:**
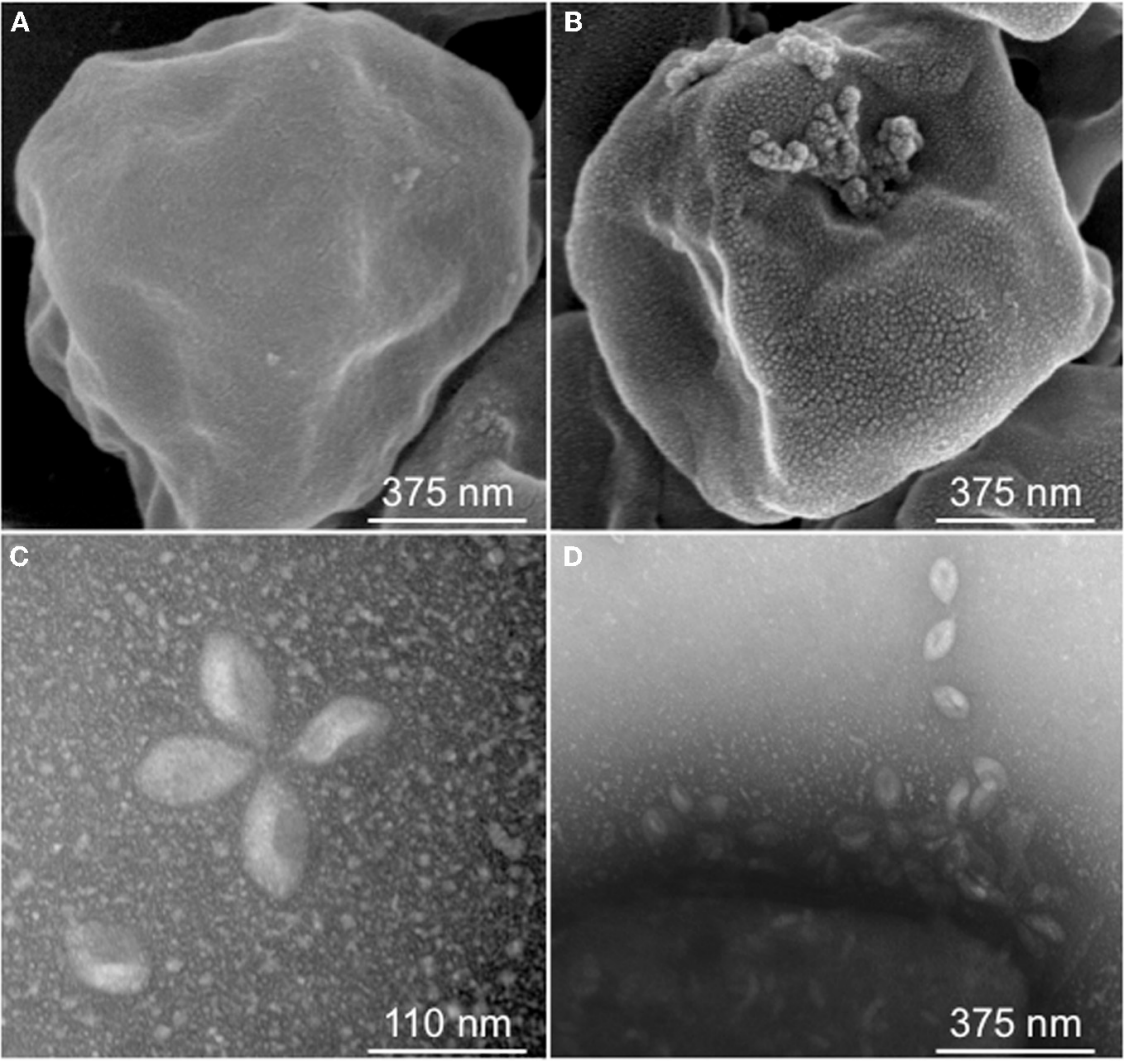
***Sulfolobus* Spindle-shaped Virus (SSV) infection.** Scanning Electron Micrograph (SEM) of *Sulfolobus solfataricus* strain Gθ either uninfected **(A)** or infected with SSVRH **(B)**; **(C)** Transmission Electron Micrograph (TEM) of typical SSVRH virions used to infect *Sulfolobus solfataricus* strain Gθ; **(D)** TEM of SSVRH virus particles with infected *Sulfolobus solfataricus* strain Gθ (lower left of image).

An emerging model virus-host system is that of the *Fuselloviridae* and their hyperthermophilic archaeal hosts (Schleper et al., 1992; Frols et al., 2007; Stedman, 2008; Redder et al., 2009). Many of the hyperthermophilic Archaea are members of the Kingdom Crenarchaea. The best-studied of these are from the family *Sulfolobaceae*, which consists of the genera *Sulfolobus*, *Acidianus*, *Metallosphaera*, *Stygiolobus*, *Sulphurisphaera*, and *Sulfurococcus* (Huber and Stetter, 2001). These organisms are found worldwide in volcanic hot springs and grow optimally at temperatures between 60 and 90°C with optimal pH from 2 to 3 but can also be found in more diverse environments. An amazing array of novel viruses that infect *Sulfolobus* and *Acidianus* have been discovered, resulting in the introduction of an unprecedented seven new virus families (Prangishvili et al., 2006). The first-discovered and best-studied of these is the virus family *Fuselloviridae* (Stedman, 2008). The original and type strain is *Sulfolobus* Spindle-shaped Virus 1 (SSV1), isolated from the host *Sulfolobus shibatae* strain B12 from geothermal springs in Beppu, Japan (Yeats et al., 1982; Martin et al., 1984). Fully-assembled fusellovirus virions typically have a “lemon” or “spindle” shape with major and minor axes of approximately 90 nm by 60 nm (see Figure 1). Short tail fibers extend from the end of one major axis (Martin et al., 1984; Stedman, 2008). The fusiform (spindle-shape) morphology is unique to the archaeal viruses for reasons that are currently unclear (Prangishvili et al., 2006).

Fuselloviruses, or viruses morphologically similar to them, have been found in up to 8% of acidic hot springs in Iceland (Zillig et al., 1998) and are the most common viruses found in sulfuric geothermal systems (Stedman, 2008). Fuselloviruses contain circular double-stranded DNA genomes that vary in size from 14–17 kbp [21 kbp for *Acidianus* Spindle-shaped Virus (ASV1)] and are found either episomally, integrated in the host genome as proviruses, or packaged as fully-assembled virions. To date, eleven “free” fuselloviruses have been reported in the literature: SSV1 (a.k.a. SAV1) (Martin et al., 1984; Palm et al., 1991), SSV2 (Stedman et al., 2003), SSV3 (Stedman et al., 2006), SSV4 (Peng, 2008) SSV5 (Redder et al., 2009), SSV6 (Redder et al., 2009), SSV7 (Redder et al., 2009), SSVK1 (Wiedenheft et al., 2004), SSVRH (Wiedenheft et al., 2004), SSVL1 (Clore, unpublished), and an *Acidianus* virus, ASV1 (Redder et al., 2009). Here, we define “free viruses” to include genomes derived from fully-assembled virions and episomal viral genomes as opposed to integrated proviruses. Recent sequencing of geographically diverse *Sulfolobus* genomes allowed the identification of four additional SSV proviruses (Held and Whitaker, 2009). For all fuselloviruses, a core set of 13–14 genes, of about 35, are conserved. However, most SSV-genomes have greater than 20 SSV-specific conserved genes as well as syntenic genomic organization (Stedman et al., 2003; Wiedenheft et al., 2004; Held and Whitaker, 2009; Redder et al., 2009).

Most fusellovirus studies have reported the discovery of the virus, its morphology, the genome, and the host from which it was isolated. However, no comprehensive host-range study has been undertaken. Given that many of these fuselloviruses and their hosts are derived from geographically-distinct geothermal regions and that previous reports suggest that there are differences in viral performance between strains (Schleper et al., 1992; She et al., 2001), such host-range studies are critical for understanding the biology of this virus-host system.

Limited host-range studies have been performed with SSV1 but none have been reported for other SSV strains. SSV1 was shown to exhibit a narrow host range in that it infects *Sulfolobus solfataricus* strains P1 and P2 (two isolates from Piscarelli, Italy) but not *Sulfolobus acidocaldarius* (Schleper et al., 1992). Subsequently, a shuttle-vector constructed from SSV1, pKMSD48, was shown to infect the *Sulfolobus solfataricus* Gθ strain, a fast-growing derivative of *S. solfataricus* strain MT3, which was also isolated from Italy (Cannio et al., 1998; Stedman et al., 1999; Cannio et al., 2001). However, for most of the other *Sulfolobus* Spindle-shaped Viruses (SSVs), infection has only been shown for *S. solfataricus* strain P2 (She et al., 2001). A further limitation in the characterization of fusellovirus-host systems is that much of the information regarding SSV production has only been described anecdotally. Specifically, no comparative “one-step growth curves” have been published and minimal data are available regarding SSV performance under varied conditions of pH and temperature, both of which are known to occur in their natural environments.

Conspicuously lacking in the literature is an examination of host-range for SSVs isolated from geographically-distinct geothermal regions. This gap is surprising considering that *Sulfolobus* has been shown to exhibit biogeographic structure in its distribution (Whitaker et al., 2003; Grogan et al., 2008). The extent to which SSVs and other crenarchaeal viruses share the same biogeography as the *Sulfolobus* host is controversial. Two reports based on virus and provirus sequences (Stedman et al., 2006; Held and Whitaker, 2009) suggest virus endemism and biogeographical separation. A study based on culture-independent environmental DNA sequence analysis suggests that SSV-like viruses are globally-distributed (Snyder et al., 2007). Moreover, high partial DNA sequence similarity between different isolated SSVs from the same geographical locations also indicated global spread of these viruses (Redder et al., 2009). However, when considering whole genome sequences or concatenations of multiple loci for free virus and provirus, there appears to be geographic separation between SSV genotypes (Held and Whitaker, 2009).

Because *Sulfolobus* strains are dispersal-limited, it is reasonable to expect that SSV infectivity profiles would reflect host biogeographic structure, especially if these parasites specialize on local hosts as a result of tight virus-host coevolutionary dynamics. In this study, spot-on-lawn halo assays (Schleper et al., 1992; Stedman et al., 2003) are used to determine host ranges for six *bona fide* SSVs, including: SSV1 (Kyushu, Japan); SSV2 (Reykjanes, Iceland); SSV3 (Krisovic, Iceland); SSVK1 (Kamchatka, Russia); SSVRH (Yellowstone National Park, MT/WY, USA); and, a recent isolate, SSVL1 (Lassen Volcanic National Park, CA, USA). These SSVs along with a set of 13 Sulfolobaceae were used to complete a comprehensive host range study, which includes a simple test to determine if SSVs infect beyond the genus *Sulfolobus*. To evaluate the stability of SSV infectivity under variations in environmental conditions that might be encountered in natural habitats, assays were also performed with varying temperature and pH.

## Materials and methods

### Host growth and virus preparation

Glycerol stocks of SSV-infected *Sulfolobus* strains stored at −80°C were partially thawed on ice. 10–20 μL of stock was added to a flask with 30 mL of media and culture was placed in a shaking water bath set at 78–80°C/70 RPM. Yeast-Sucrose (YS) media (1X) was used for most inoculations; however, for strains that do not grow well in YS media a tryptone-based media (1X) was used instead (Stedman et al., 2003). Once the seed culture reached an optical density (OD_600_) between 0.4 and 0.6, 3.0 mL of cell suspension was used to inoculate 300 mL of pre-heated fresh medium and cultures were, again, incubated at 78–80°C/70 RPM until reaching an OD_600_ = 0.4–0.6, at which time virus was harvested. Harvesting virus consisted of centrifuging all 300 mL of the culture for 20 min at 6000 RPM (Sorvall RT Legend Centrifuge, Fiberlite fixed-angle rotor; ThermoFisher, Pittsburgh, PA). The supernatant was decanted and filtered through a 0.45 μm filter and 300 mL of filtrate was concentrated to ~1.5 mL using a Centricon Plus-70 spin concentration tube (Millipore Corp., Billerica, MA, USA). Transmission electron microscopy (TEM) was used to confirm the presence of SSV-like virions (described below). Virus concentrates were stored at 4°C and used within 2–3 weeks of harvesting.

### Spot-on-lawn infection assays

Infectivity and susceptibility assays were performed essentially as in (Stedman et al., 2003). Uninfected cultures were grown as described above. 500 μL of OD_600_ = 0.4–0.6 culture was added to 4.5 mL of 0.25% w/v Gelrite® (Sigma-Aldrich, St. Louis, MO) in YS medium at 78°C. The mixture was spread on pre-warmed 1% w/v Gelrite plates, allowed to solidify for 15 min at RT, then incubated for 20 min at 78°C. After this short incubation period, 1.0 μL of virus suspension was spotted onto the lawns and plates were incubated for 3–9 days at 78°C (3–6 replicates per set typical). Plates were checked daily for up to 9 days. Inhibition of host cell growth, as evidenced by a visible halo of growth inhibition, was scored as a successful infection. Triton X-100 (0.01% v/v) was used as a positive control and sterile water as a negative control.

### Transmission electron microscopy (TEM)

Approximately 5 μL of viral suspension was spotted onto a formvar-coated copper grid and incubated for 10 min in a humidity chamber. The grid was rinsed with distilled water and negatively stained with 1% (w/v) uranyl acetate for 30 s. The stain was wicked off and the sample was air dried. Grids were imaged in a Hitachi H-7100 TEM at 75 kV. Virus images were captured at 60,000–150,000× magnification. Note that these preparations were spin-filtered and concentrated viral suspensions, not scraped plaques.

### Scanning electron microscopy (SEM)

Cell suspensions were centrifuged (4000 RPM for 15 min), the supernatant was decanted, the cell pellet was resuspended in sodium cacodylate buffer (pH = 6) with 5% (v/v) EM-grade glutaraldehyde, and the suspension was left overnight at 4°C. Fixed cells were washed in distilled water and fixed in 2% osmium tetroxide for 2 h at 4°C, washed twice in distilled water, and resuspended in dH_2_O prior to ethanol dehydration. An aliquot of cell suspension was placed on a 0.6 mm isopore membrane filter using a syringe and then dehydrated using a graded ethanol series of 35, 50, 70, 90, 95, and 100% (v/v). This dehydration step was repeated once. Following the second 100% ethanol step, the filter was placed in 100% hexamethyldisilazane (HMDS) for 30 min then air-dried. The filter was sputter-coated with gold/palladium and imaged in a Hitachi S-4700 scanning electron microscope.

### Plate imaging—technical notes

For some virus-host pairings, halos were very faint and could only be viewed by holding the plate in front of and close to a light source. Flash photography of faint halos proved difficult. SSV1 and SSVL1 in particular produced faint halos. In some cases, host growth was not robust; this also resulted in faint halos. Some lawns were damaged on one section of the plate, typically by condensation. In these instances the section of the plate that could be scored was scored. The damaged section of the plate was excluded from analyses. This issue was particularly problematic in the low pH (pH < 2.0) trials. Adding additional Mg^++^ and Ca^++^ ions produced slightly more stable plates at low pH but, for most plates, there were sections that prematurely dissolved or that could otherwise not be scored. Low pH assays were performed until a minimum number of replicates for each host-virus pairing could be scored. Gelrite®-plates at pH > 2.0 typically maintain soft-layer and base integrity for up to 9 days at 78–80°C. Separating stacked plates with rubber risers improved stability by distributing hot air flow.

## Results

### All SSVs tested formed halos of growth inhibition on *S. solfataricus* strain Gθ lawns

**Figure 2:**
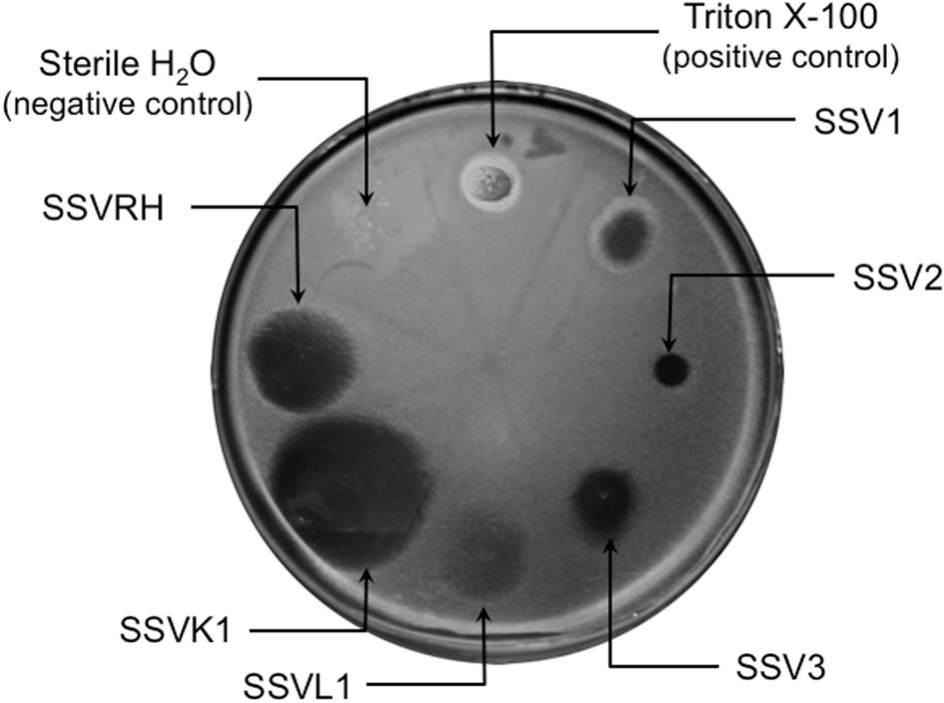
**SSV-strain specific halo formation on *Sulfolobus solfataricus* strain Gθ by all six SSV tested strains.** Virus susceptibility is determined by the production of halos of growth inhibition around a 1.0 μL spot of virus concentrate applied to a Gelriteθ softlayer containing *S. solfataricus* strain Gθ. Locations of spots and identities of SSVs are indicated with arrows. Plate were incubated for 4 days at 78°C. Halo morphologies are typical for each SSV-strain. Triton X-100 and sterile H_2_O serve as positive and negative controls, respectively.

Fifteen full fusellovirus genome sequences and at least two virus-free host genome sequences have been determined. However, only anecdotal mention of between-strain differences in SSV infection properties has been reported (Palm et al., 1991; She et al., 2001; Stedman et al., 2003; Wiedenheft et al., 2004; Held and Whitaker, 2009; Redder et al., 2009). To date, all of the virus-producing *Sulfolobus* strains have been identified by their ability to form halos of growth inhibition or plaques on lawns of virus-free isolates of *S. solfataricus* strains P1 and/or P2, or closely-related “*S. islandicus*” strains, or by direct observation of culture supernatants (Stedman, 2008; Redder et al., 2009). The exception to this is a recent report of a novel fusellovirus, the ASV1 found in *Acidianus brierleyi* (Redder et al., 2009). Here we directly compare the infection capabilities of six geographically-distinct SSV strains: SSV1 (Kyushu, Japan), SSV2 (Reykjanes, Iceland), SSV3 (Krisovic, Iceland), SSVL1 (California, USA), SSVK1 (Kamchatka, Russia), and SSVRH (Wyoming, USA)—by spot-on-lawn or “halo” assays (Stedman et al., 2003) against multiple Sulfolobales strains. In order to obtain robust host controls all six SSVs were tested on three Italian strains of *Sulfolobus solfataricus*, a known host for many SSVs (Schleper et al., 1992; Stedman et al., 1999, 2003; Wiedenheft et al., 2004). All three of these Italian strains were susceptible to all six tested SSVs. Halos of growth inhibition on lawns of *S. solfataricus* strain Gθ were observed on a single plate (*n* = 9; three trials, each in triplicate) for all six SSV strains tested (Figure 2). Since *S. solfataricus* Gθ (Cannio et al., 1998) generally grew faster than *S. solfataricus* strains P1 and P2, strain Gθ was selected as the host for control plates, which were run in parallel with other Sulfolobales host lawns (thus, the higher number of repetitions *n* for Gθ in Table 1). Triton X-100 served as a technical positive control as it consistently formed an area of complete host-growth suppression. Sterile water served as a negative control. As an additional control we performed TEM and SEM on uninfected and infected cultures (Figure 1). Moreover, in order to confirm the identity of viruses after plaque assays, we performed PCR on virus genomes with SSV-specific primers (data not shown).

**Table 1:** **Infectivity/susceptibility profiles**.

	**Host strain**		**Virus**						
**Loc**	**Ref**	**Description**	**SSV1**	**SSV2**	**SSV3**	**SSVL1**	**SSVK1**	**SSVRH**	**S**
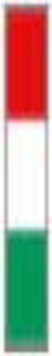	Cannio et al., 1998	*Sulfolobus solfataricus* Gθ (biological positive control)	+^a^	+	+	+	+	+	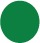
			(*n* = 30)^b^	(*n* = 27)	(*n* = 28)	(*n* = 23)	(*n* = 32)	(*n* = 24)	
	DSM 1616	*Sulfolobus solfataricus* P1	+	+	+	+	+	+	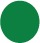
			(*n* = 9)	(*n* = 9)	(*n* = 9)	(*n* = 6)	(*n* = 9)	(*n* = 6)	
	DSM 1617	*Sulfolobus solfataricus* P2	+	+	+	+	+	+	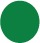
			(*n* = 6)	(*n* = 3)	(*n* = 4)	(*n* = 4)	(*n* = 6)	(*n* = 6)	
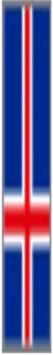	Zillig et al., 1994	*Sulfolobus islandicus* REN1H1 (with pRN1, pRN2 plasmids)	−	−	−	−	−	−	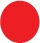
			(*n* = 5)	(*n* = 5)	(*n* = 4)	(*n* = 5)	(*n* = 4)	(*n* = 6)	
	Prangishvili et al., 2006	*Sulfolobus islandicus* REN1H1	−	−	−	−	−	+	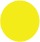
			(*n* = 2)	(*n* = 4)	(*n* = 2)	(*n* = 2)	(*n* = 2)	(*n* = 6)	
	Peng, 2008	*Sulfolobus islandicus* HVE10/4	−	+	+	−	+	+	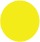
			(*n* = 4)	(*n* = 4)	(*n* = 4)	(*n* = 5)	(*n* = 6)	(*n* = 6)	
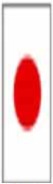	Stedman et al., 1999	*Sulfolobus tokodaii*	−	−	−	−	−	−	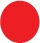
			(*n* = 4)	(*n* = 5)	(*n* = 6)	(*n* = 8)	(*n* = 6)	(*n* = 7)	
	Keeling et al., 1998	*Sulfurisphaera ohwakuensis*	−	−	+	−	+	+	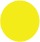
			(*n* = 5)	(*n* = 5)	(*n* = 4)	(*n* = 7)	(*n* = 5)	(*n* = 6)	
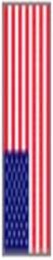	DSM 639	*Sulfolobus acidocaldarius*	−	−	−	−	−	−	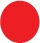
			(*n* = 3)	(*n* = 3)	(*n* = 3)	(*n* = 3)	(*n* = 3)	(*n* = 3)	
	(SU)^c^	*Sulfolobus sp*. (Yellowstone NP)	−	+	+	+	+	+	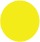
			(*n* = 4)	(*n* = 4)	(*n* = 4)	(*n* = 7)	(*n* = 4)	(*n* = 4)	
	(SU)	*Sulfolobus sp*. (Lassen NP)	+	+	+	+	+	+	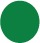
			(*n* = 6)	(*n* = 6)	(*n* = 6)	(*n* = 6)	(*n* = 6)	(*n* = 6)	
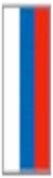	(SU)	*Sulfolobus sp*. (Kamchatka GV)	−	+	+	+	+	+	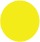
			(*n* = 4)	(*n* = 4)	(*n* = 4)	(*n* = 4)	(*n* = 4)	(*n* = 4)	
	(SU)	*Sulfolobus sp*. (Kamchatka MU)	−	−	−	−	−	−	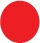
			(*n* = 4)	(*n* = 4)	(*n* = 4)	(*n* = 4)	(*n* = 4)	(*n* = 4)	

Loc, Location of isolation (from top to bottom): Italy, Iceland, Japan, USA, Russia.

S, Susceptibility: completely susceptible (green), selectively resistant (yellow), completely resistant (red).

a “+” indicates halo formation and productive virus infection; “-” indicates no detectable halo.

b n, number of clearly observed halos for “+” and number of independent trials for “-”.

c SU, Stedman, unpublished.

### Different SSVs produce different halo morphologies when infecting *S. solfataricus* Gθ

Although halos of growth inhibition on lawns of *S. solfataricus* strain Gθ were observed on a single plate for all six SSVs tested, the morphology of the halos differed between viruses (Figure 2). SSV1 consistently produced a turbid halo with very diffuse edges and often featured a secondary ring. This diffuse double-ring morphology differed from SSV2, which produced a clear single-ringed halo with sharp edges, and from SSV3, which produced a single-ring halo with diffuse edges. SSVL1 produced faint turbid halos of moderate size. Both SSVK1 and SSVRH produced clear halos with well-bounded edges. SSV strain-specific halo morphologies were consistent across different virus preparations (*n* = 4). Differences in halo size were found to be a function of spotted virus concentration (data not shown). SSV strain-specific halo morphologies persisted regardless of the concentration of the viral suspension used. *Sulfolobus*-SSV cell-virus interaction was confirmed using SEM and TEM (Figure 1). The spot-on-lawn approach is the optimal high-throughput method for these assays since it allows direct comparison between multiple viruses on a single lawn (and duplicates on the same plate).

### SSVs exhibit different host-ranges even among closely-related *Sulfolobales* strains

**Figure 3:**
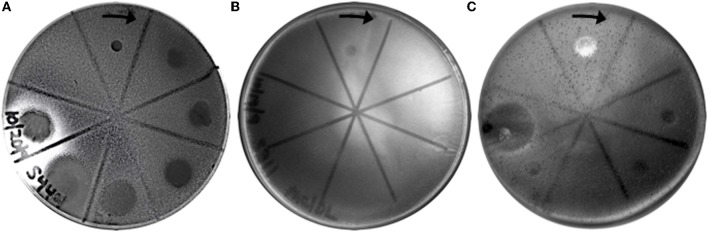
**Representative virus susceptibility of the three host classes.** Virus infection was determined as in Figure 2. **(A)** *Completely susceptible host:* All 6 SSVs tested inhibit growth of a *Sulfolobus* strain isolated from Lassen Volcanic National Park. **(B)** *Completely resistant host*: No SSVs inhibit growth of *S. tokodaii*. **(C)** *Selectively susceptible host*: 4 of 6 SSV strains inhibit the growth of “*S. islandicus*” strain HVE 10/4. Spots on plates are (*clockwise from arrow*): Triton X-100 (positive control), SSV1, SSV2, SSV3, SSVL1, SSVK1, SSVRH, and sterile H_2_O (negative control). The complete dataset for host-virus interactions is shown in Table 1.

*Sulfolobus* species have been reported to have considerable variability within the genus and even between strains of the same species (Grogan, 1989; Reno et al., 2009). Therefore, SSV1, SSV2, SSV3, SSVL1, SSVK1, and SSVRH were screened for infectivity on 12 strains of *Sulfolobus* (including the positive control strain Gθ) plus the closely-related crenarchaeon *Sulphurisphaera ohwakuensis*. Susceptibility to SSV infection for each potential host varied. Some were susceptible to all six SSVs tested (Figure 3A), some were completely resistant to SSV infection (Figure 3B), and others were susceptible to only a subset of the six SSVs tested (Figure 3C). Host-range data for all virus-host combinations tested are shown in Table 1.

#### Completely susceptible

Although susceptibility of some of these *Sulfolobus* strains to SSV infection had previously been reported (Schleper et al., 1992; Stedman et al., 2003), no previous study tested them simultaneously and in direct comparison to each other under identical growth conditions. All three strains isolated from hot springs in Italy: *S. solfataricus* strains P1, P2, and Gθ—are viable hosts for all six SSV strains tested. Notably, complete susceptibility to all SSVs tested was not limited to hosts isolated from Italy. In addition, a novel *Sulfolobus* isolate from Lassen Volcanic National Park in California (Clore, unpublished) also was susceptibile to infection by all SSVs tested (Table 1; Figure 3A).

#### Completely resistant

Previous studies demonstrated that *S. acidocaldarius* isolated from Yellowstone is resistant to SSV1 and SSV2 (Schleper et al., 1992; Stedman et al., 2003). This study confirms these previous results and demonstrates that *S. acidocaldarius* is additionally resistant to the other four SSVs tested in this study. Three other strains also exhibited complete resistance to the SSVs used in this study: *S. tokodaii* (Figure 3B) from Beppu, Japan (Suzuki et al., 2002), a Russian *Sulfolobus* strain isolated from the Mutnovsky volcanic region of Kamchatka (Stedman, unpublished), and a Icelandic strain, “*S. islandicus*” REN1H1 which harbors plasmids pRN1 and pRN2 (Zillig et al., 1994).

#### Selectively resistant

**Figure 4:**
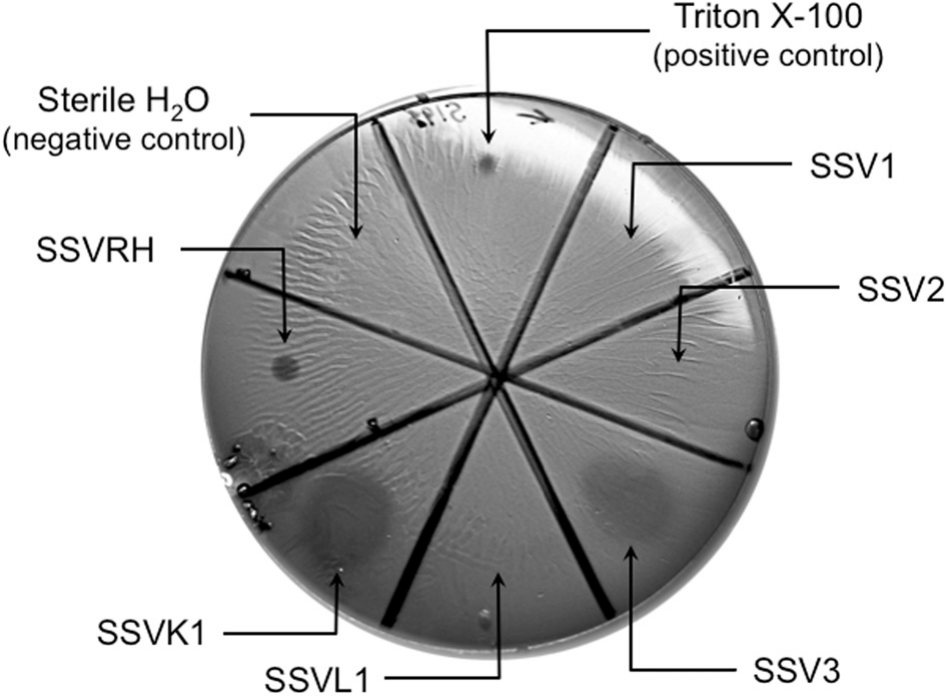
***Sulphurisphaera ohwakuensis* is susceptible to SSV infection.** Virus infectivity was assessed as in Figures 2 and 3. SSV3, SSVRH, and SSVK1 produce halos on *Sulphurisphaera ohwakuensis*, a Japanese isolate, closely related to *Sulfolobus tokodaii*. SSV1, SSV2, and SSVL1 did not inhibit growth of this strain.

Many *Sulfolobus* strains were neither completely susceptible nor completely resistant to the six SSVs tested but instead were susceptible to a subset of tested SSVs. Moreover, many of these “selectively resistant” hosts exhibited different susceptibility profiles. *Sulfolobus islandicus* strain HVE10/4 from Hveragerdi, Iceland (Prangishvili et al., 1999) was resistant to infection by SSV1 from Japan and SSVL1 from Lassen Volcanic National Park, USA but was susceptible to infection by: SSV2 from Reykjanes, Iceland; SSV3 from Krisovic, Iceland; SSVRH from Yellowstone National Park, USA; and SSVK1 from Kamchatka, Russia. *Sulfolobus islandicus* strain REN1H1, which harbors two plasmids pRN1 and pRN2, neither of which have sequence similarity to any SSV genomes, was resistant to all SSVs. Yet, an REN1H1 strain lacking the plasmids (Purschke and Schafer, 2001) was susceptible to infection by SSVRH. Although *Sulfolobus tokodaii* exhibited resistance to all SSVs tested, the genomically similar *Sulphurisphaera ohwakuensis* (Kurosawa et al., 1998) was susceptible to infection by SSV3, SSVK1, and SSVRH (Figure 4). This is the first report of any SSV establishing infection beyond the genus *Sulfolobus* and demonstrates that susceptibility to SSV infection is not necessarily a function of ribosomal DNA (16S) relatedness. Both susceptible and completely resistant strains are in closely-related clades in *Sulfolobaceae*. For example, *Sulfolobus tokodaii* and *Sulfurisphaera ohwakuensis* have identical 16S rDNA genes (>99%) and *S. tokodaii* and *S. acidocaldarius* have 90% 16S rDNA genes, yet the latter pair has the same lack of susceptibility to SSV infection (Table 1) while the former pair has differential susceptibility. This further supports the suggestion that susceptibility to SSV infection may be driven by specific genomic features rather than overall genetic divergence (discussed below).

### Growth inhibition by SSV infection is temperature independent between 65°C and 88°C, but appears to be affected by low pH

Halo assays for the host range studies (Figures 1, 3, 4 and Table 1) were performed under optimal temperature and pH conditions for *Sulfolobus* growth (78–80°C, pH = 3.0–3.2). However, not all of these strains were isolated from environments with these conditions. Moreover, hydrothermal environments are chronically variable. SSV1 is reported to maintain activity for at least 45 min at 89°C and pH 5.5; however, the SSV1 genome is reported to be unstable at low pH, even though the virions appear to remain intact (Schleper et al., 1992). We found that SSVK1 remains infectious after exposures to solutions with pH values of 1–11 for 1 h at room temperature (Morris and Stedman, unpublished). However, no information regarding virus stability and infectivity at various temperatures and pH is published for any of the other SSVs used in this study. Thus, different temperature and pH conditions were tested to determine if infectivity was affected. Although all possible pairings were not tested, several SSV-*Sulfolobus* pairs were used in these assays. The most rigorously tested host strain was *S. solfataricus* strain Gθ (see Table 2). Host lawn and halo growth *rate* did vary between tests (slower at 65°C) but neither incubation at low temperature (65°C) nor at high temperature (88°C) qualitatively changed infectivity relative to the control (78°C). These data suggest that qualitative SSV infectivity and host susceptibility is stable at different temperatures. Separate preparations of media and plates with pH values of 1.2, 2.2, 3.2, and 4.2 were then used to culture cells and grow lawns of *S. solfataricus* Gθ. All plates were incubated at 78–80°C. After three trials (each in triplicate), all six SSVs readily inhibited *S. solfataricus* Gθ growth on plates prepared at pH = 3.2 and 4.2 (Table 2). At lower pH values, plate integrity was variable and many plates collapsed despite robust host growth. In spite of these technical issues, several halos were detected for each SSV strain at pH = 2.2. Yet, at the lowest pH (1.2) only SSV1, SSV2, and SSVL1 formed any halos, suggesting that very low pH can affect SSV infectivity.

**Table 2:** **Halo formation on *S. solfataricus* Gθ Lawns at differing pH values**.

**Virus**	**pH of Media (3 trials in triplicate)**				
	**pH = 1.2^a^**		**pH = 2.2**	**pH = 3.2 (control)**	**pH = 4.2**
SSV1	+	−	+	+	+
	(*n* = 3)	(*n* = 3)	(*n* = 3)	(*n* = 9)	(*n* = 9)
SSV2	+	−	+	+	+
	(*n* = 3)	(*n* = 3)	(*n* = 4)	(*n* = 9)	(*n* = 9)
SSV3	−		+	+	+
	(*n* = 6)		(*n* = 2)	(*n* = 9)	(*n* = 9)
SSVL1	+	−	+	+	+
	(*n* = 3)	(*n* = 3)	(*n* = 3)	(*n* = 9)	(*n* = 9)
SSVK1	−		+	+	+
	(*n* = 3)		(*n* = 3)	(*n* = 9)	(*n* = 9)
SSVRH	−		+	+	+
	(*n* = 3)		(*n* = 3)	(*n* = 9)	(*n* = 9)

a The integrity of some plates failed at this pH.

## Discussion

### SSV-*Sulfolobus* interactions appear to be independent of geographic separation

In line with theories regarding parasite-host local adaptation (Keeling et al., 1998; Gandon and Michalakis, 2002; Greischar and Koskella, 2007), two hypotheses concerning SSV host ranges were tested in this work. The first is that local viruses are better at infecting local hosts than geographically distant hosts. The second is that local hosts should be more resistant to local viruses than distant viruses. Interestingly, in the SSV-*Sulfolobus* system, neither of these hypotheses appears to be supported.

### SSV infection of *Sulfolobus* is not dependent upon host geography

Different *Sulfolobus* strains have different SSV susceptibility profiles (Table 1) and geography does not appear to be a useful predictor of susceptibility. Potential hosts included strains from six distinct geographic regions: Iceland; Italy; Japan; Kamchatka, Russia; and Lassen Volcanic National Park and Yellowstone National Park in the USA. For example, the Icelandic host strain HVE10/4 exhibited susceptibility to four of the SSVs tested, including the two Icelandic viruses (SSV2 and SSV3), the Yellowstone virus (SSVRH), and the Russian virus (SSVK1). However, the wild-type, plasmid-containing Icelandic strain REN1H1 shows complete resistance to infection by all SSVs tested. Neither of these plasmids contains sequences similar to any SSV, so the mechanism of this resistance is unclear. In addition to the REN1H1 strain, three other *Sulfolobus* strains were resistant to infection by all SSVs tested: *S. tokodaii*, a Japanese isolate; *S. acidocaldarius* from Yellowstone National Park (USA); and an isolate from the Mutnovsky Volcano region (Russia). Nevertheless, other isolates from each of these regions were at least partially susceptible to a set of allopatric SSVs. In addition, an isolate from Lassen Volcanic National Park was found to be susceptible to all SSVs tested. In summary, hosts from all geographic regions tested (except Italy) included at least one strain that was completely resistant to all six SSVs tested as well as some that were sensitive to infection by at least one SSV. These susceptibility data are unexpected due to phylogenetic data on *Sulfolobus*, which suggest that host genetic divergence is positively correlated with geographic distance between natural habitats (Grogan, 1989; Whitaker et al., 2003; Grogan et al., 2008; Reno et al., 2009), which, in turn, might suggest that infectivity is likewise correlated to geography.

### Infectivity profiles of SSVs are also not geography dependent

Similar to host susceptibility, different SSVs have different infectivity profiles (Table 1). These viruses were isolated from five distinct geographic regions: Iceland; Japan; Kamchatka, Russia; and Lassen Volcanic National Park and Yellowstone National Park in the USA. SSV1 from Japan has the narrowest host range, whereas SSVRH from the USA has the broadest. SSVK1 from Russia and SSV3 from Iceland infected all of the same hosts as SSVRH with one exception, plasmid free- *S. islandicus* REN1H1. However, even SSV1, which exhibited the narrowest host-range, was able to infect all three Italian isolates, indicating that geographic separation of host strains and virus isolation does not limit infection. Thus, similar to host susceptibility, geography is not a reliable predictor of SSV infectivity. Independent data from SSV sequences amplified from Yellowstone hot springs suggest that these viruses are globally mobile (Snyder et al., 2007); however, complete genome sequence data of the six SSVs investigated here and others indicate endemism (Stedman et al., 2006; Held and Whitaker, 2009) and high between-strain genetic similarity (Stedman et al., 2003; Wiedenheft et al., 2004; Redder et al., 2009). The molecular basis for these different infectivity profiles remains to be determined.

### SSV infection is not host species dependent

*Sulfolobus acidocaldarius* has already been shown to be resistant to infection by SSV1 and SSV2 (Schleper et al., 1992; Stedman et al., 2003). Here we show that it is also resistant to SSV3, SSVL1, SSVRH, and SSVK1. Similarly, *S. tokodaii* is also completely resistant to all SSVs tested. Surprisingly, *Sulfurisphaera ohwakuensis* (Kurosawa et al., 1998), a species described as being a different genus but with a small subunit rDNA sequence greater than 99% identical to *S. tokodaii* (Suzuki et al., 2002), was susceptible to infection by both SSVRH and SSVK1. These results led us to test other closely-related *Sulfolobus* strains. *S. islandicus* strain REN1H1 naturally harbors two plasmids—pRN1 and pRN2 (Zillig et al., 1994; Keeling et al., 1998). This strain is completely resistant to all SSVs tested. *Sulfolobus islandicus* strain REN1H1 has been cured of both plasmids (Purschke and Schafer, 2001) and the resulting strain is susceptible to SSVRH. Whether this susceptibility is due to lack of plasmids is unclear.

### Infection resistance does not correlate with CRISPR spacer matches

The recently characterized CRISPR/Cas system, present in most archaeal and many bacterial genomes, is proposed to be involved in acquired resistance to virus infection (Horvath and Barrangou, 2010). This has been clearly demonstrated for *Streptococcus thermophilus* (Barrangou et al., 2007) but has yet to be conclusively demonstrated for Archaea. Acquired virus resistance is due to the presence of a matching “spacer” sequence in a host CRISPR locus. Therefore, we checked the genomes of *S. solfataricus* strain P2 (She et al., 2001), *S. acidocaldarius* (Chen et al., 2005) and *S. tokodaii* (Kawarabayasi et al., 2001)—the three tested hosts for which complete genomes are available—for sequence matches to the six tested SSVs. Of all six SSVs and genomes, only SSV1 matched an annotated CRISPR spacer in the *S. tokodaii* genome with 41 basepairs with one mismatch. However, *S. tokodaii* is resistant to infection by all SSVs, not just SSV1 (Table 1). Moreover, *S. acidocaldarius*, with no CRISPR matches to SSVs, is also completely resistant to SSV infection (Table 1). This indicates that the resistance of *S. tokodaii* and *S. acidocaldarius* may be independent of the CRISPR/Cas system. We cannot exclude that the resistance of selectively resistant strains is due to the CRISPR/Cas system, thus genome sequencing of some of these hosts is underway. Other infection resistance mechanisms include regulation of adsorption, other aspects of DNA entry, genome replication, transcription, translation, assembly, or virus release (Labrie et al., 2010). Very little is known about any of these mechanisms for the *Fuselloviruses*.

### SSV infectivity is not dependent on temperature but is influenced by pH

Both pH and temperature fluctuations are known to occur in *Sulfolobus* habitats (Snyder et al., 2007). SSV1 has been shown to be stable for 45 min at 89°C and pH 5.5 after which it remained infectious (Schleper et al., 1992). Here we extend these data and show that SSV1 and other SSVs are infectious at both higher and lower temperatures for 3–5 days in halo assays. However, varying pH within the physiological range caused changes in infectivity. At lower pH values, SSV3, SSVK1, and SSVRH were unable to readily form halos, whereas SSV1, SSV2, and SSVL1 occasionally did (Table 2). It is unknown whether the observed decreases in infectivity under more acidic conditions are due to more rapid virus degradation at lower pH, changes in virus-host interactions, or a combination of these or other factors. The SSV1 virus genome is reported to be unstable at low pH, but the virus particle appears to be stable (Schleper et al., 1992).

### There is no evidence for local adaptation in the SSV-*Sulfolobus* virus-host relationship

SSV-*Sulfolobus* host-range experiments were also designed to test both “home vs. away” (single virus and multiple hosts) and “foreign vs. local” (multiple viruses on a single host) criteria for virus local adaptation (Kawecki and Ebert, 2004). Under the assumption that SSVs have high migration rates (Snyder et al., 2007) relative to their hosts, local adaptation is expected (Greischar and Koskella, 2007). However, we see no evidence for qualitative virus local adaptation (Table 1). Qualitative virulence differences between SSVs are apparent in different halo morphologies between different viruses (Figure 1). Quantitative fitness measurements for virus-host pairs are underway.

Provided that virus migration is equal to or greater than that of the host, it is predicted that viruses possess a greater evolutionary potential than their hosts due to their rapid replication rates and large populations sizes (Greischar and Koskella, 2007). Thus, in a structured system either a universally virulent virus genotype will emerge or a geographic gradient is expected along which infectivity changes as a function of geographic separation (Gandon and Michalakis, 2002). To the contrary, our data indicate that some *Sulfolobus* strains are universally resistant to SSV infection (Table 1). The presence of a CRISPR spacer match to SSV1 in the *S. tokodaii* genome indicates that at least *S. tokodaii* was susceptible to SSV infection at some point, eliminating a trivial explanation for resistance such as lack of virus receptor. Since *S. tokodaii* appears to have acquired resistance to SSV infection, it must have gained an advantage in the SSV-*Sulfolobus* “coevolutionary arms race.” Although SSVRH, SSVK1, and SSV3 do exhibit broad host ranges, there is no universally infectious SSV in the set of viruses that we tested. Therefore, the SSV-*Sulfolobus* system currently appears to feature host advantage.

## Conclusions

Our results indicate that: (1) Different SSV strains have different host ranges, at least three of which extend beyond the genus *Sulfolobus*, and different *Sulfolobus* strains exhibit different susceptibility to SSV infection; (2) In spot on lawn assays different SSVs elicit different halo morphologies during replication; (3) Variations in both pH and host (similar to those found in environments in which SSVs are found) have qualitative effects on SSV infection but temperature does not; (4) SSV1 exhibits the narrowest host range of all SSVs tested and SSVRH has the broadest host range; (5) SSV infectivity and *Sulfolobus* susceptibility are independent of the geographic regions from which the hosts and viruses were isolated. These results form a solid foundation to support future work focused on determining both the genetic and molecular basis for these host-range differences as well as also for quantitative studies of local adaptation in this tractable model system.

### Conflict of interest statement

The authors declare that the research was conducted in the absence of any commercial or financial relationships that could be construed as a potential conflict of interest.
